# Geographic Data on Health Inequities: Understanding Policy Implications

**DOI:** 10.1371/journal.pmed.0030357

**Published:** 2006-09-12

**Authors:** Gregory Pappas

## Abstract

Pappas discusses a new study that examines US health disparities using county-level data.

In a study in *PLoS Medicine*, Murray and colleagues take a new look at health disparities using county-level data [1]. Their findings have implications for understanding the social geography of health and for setting public health policy.

## The New Study

In the United States, there are large disparities in life expectancy between counties; an analysis of life expectancy by the combination of race and county (“race-county”) shows even wider disparities [2]. Murray and colleagues investigated the possible causes of the race-county mortality disparities by dividing race-counties into eight subgroups (Table 1), which they call the “eight Americas,” based on a number of sociodemographic and geographic variables.

**Table 1:**
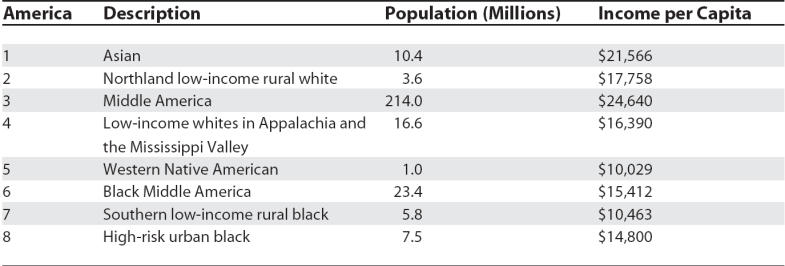
The Eight Americas, as Defined by Murray and Colleagues

The authors found some striking health disparities among the eight Americas. For example, the life expectancy gap between the 3.4 million high-risk urban black males and the 5.6 million Asian females was 20.7 years in 2001. The mortality disparities among the eight Americas were largest for young (15–44 years) and middle-aged (45–59 years) adults, especially for men. These disparities were mostly explained by a number of chronic diseases and injuries with well-established risk factors, including alcohol; tobacco smoking; overweight and obesity; and elevated blood pressure, cholesterol, and glucose.

## Limitations of a Geographic Approach

The classic limitation of the geographic approach, used by Murray and colleagues, is the assumption of homogeneity within spatial units (in this case, race-counties). We tend to be like our neighbors, but how alike are we? The major methodological weakness of the eight Americas approach is that one of the Americas (America 3, Middle America) is huge compared with the others and it is very likely that large disparities exist *within* that America.

However, I don't want to dwell on the methodological problems of geographic information. Instead, I am interested in the limitations implicit in these approaches related to their use in policy. How can an understanding of the relationships between place and health inform policy? What are the limitations of this “social geography” approach?

The appeal of social geography to health policy makers is that priorities can be attached to place. Funds can rationally flow to the neediest areas; attention can be focused on diseased locations. Targeting of health services is another policy that is well supported by geographic analysis. Information about locales also supports social marketing (i.e., using marketing messages to promote healthy behaviors, such as condom use to prevent sexually transmitted infections). Understanding of the “market” of adverse health behavior has been greatly enhanced with geographic information. Strategies to promote behavior change can be improved with good information. These policies have indeed been a mainstay of public health. These approaches focus on individuals who require services or are to be influenced. In this social geography, location is a proxy for the individual.

But while this geographic approach to health data is popular and intuitively appealing, the approach misses some critical issues and options for policies. The focus on poor individuals and poor places limits policy options. Complex social pathologies associated with modern epidemics of obesity, alcoholism, and smoking compel us to think more contextually and seek insights at the level of systems.

The results of anti-smoking campaigns may provide us with some lessons about the limitations of our efforts to tackle disparities in risk behaviors. Although there have been dramatic decreases in smoking in the US, most of the benefit has gone to the upper rungs of society. Those at the bottom continue to smoke at high rates [3]. Smokers who live in less fortunate circumstances in our society have not responded well to social marketing and targeting. Contemporary epidemics are associated with behaviors that are embedded in cultures (patterned behavior) that are more complex than individual choices. William Julius Wilson reminds us that “cultural values emerge from specific circumstances and life chances and reflect an individual's position in the class structure” [4]. Place is not only a proxy for individual characteristics but must also be understood within historic processes.

## Understanding Social Context

Targeting funding to impoverished areas or burned-out parts of cities, slums, ghettos, and barrios might not solve the health problems in the US. The social processes that lead to inequalities in health are much more complex than the identification and description of poor pockets would suggest. Health inequities are perpetuated by systemic processes that operate outside of the targeted places.

Jargowsky's now classic 1997 work, *Poverty and Place: Ghettos, Barrios, and the American City*, is still a fertile starting point to understand the root causes of health inequalities and policy solutions [5]. His work directs our attention to the ways that economic opportunity, segregation, and social policy create urban patterns of unequal places. Programs in ghettos, barrios, and slums cannot solve these problems, which are metropolitan in nature. The poverty and problems of these communities are not “self-sustaining.” Complex modern epidemics are embedded in the same social context of poverty and place. The health problems associated with poor places will not be improved simply by changing economic incentives for individuals or putting more resources near poor people.

Social and economic gaps are widening between races and classes in the US [6]. We must understand the process leading to this widening. There are fewer people living in poor places, but the disparities and the *number* of those who are poor have not decreased. Jargowsky and Sawhill recently looked at time trends in the size of underclass areas and found that the areas were shrinking [7]. Concentrated poverty is being replaced by disbursed poverty. The effort to document social inequities must be accompanied by an understanding of the social processes that underpin them, and actions to change these processes.

## Conclusion

Efforts to isolate small groups and then demonstrate health inequalities among them can produce interesting data, but these are difficult to interpret without a theoretical framework to support them. Freeman and McCord's comparison of mortality rates among men in Harlem with men in Bangladesh cannot be outdone in the simple demonstration of inequalities [8]. Murray and colleagues provide fresh methods using geographic, county-level data to explore inequalities in health [1]. However, analysis that emphasizes social process may lead us to more effective policy to address health disparities.
